# Clinical Application of Screening for *GJB2* Mutations before Cochlear Implantation in a Heterogeneous Population with High Rate of Autosomal Recessive Nonsyndromic Hearing Loss

**DOI:** 10.4061/2011/787026

**Published:** 2011-11-24

**Authors:** Masoud Motasaddi Zarandy, Mersedeh Rohanizadegan, Hojjat Salmasian, Nooshin Nikzad, Niloofar Bazazzadegan, Mahdi Malekpour

**Affiliations:** ^1^ENT Research Center, Department of Otorhinolarygology, Head and Neck Surgery, Amir Alam Hospital, Tehran University of Medical Sciences, Tehran 1145765111, Iran; ^2^Students Scientific Research Center, Tehran University of Medical Science, Tehran 1417613151, Iran; ^3^Department of Medicine, Columbia University, New York, NY 10032, USA; ^4^Department of Biomedical Informatics, Columbia University, New York, NY 10032, USA; ^5^Genetics Research Center, University of Social Welfare and Rehabilitation Sciences, Tehran, Iran; ^6^Department of Medicine, Vanderbilt University Medical Center, Nashville, TN 37232, USA

## Abstract

Clinical application of mutation screening and its effect on the outcome of cochlear implantation is widely debated. We investigated the effect of mutations in *GJB2* gene on the outcome of cochlear implantation in a population with a high rate of consanguineous marriage and autosomal recessive nonsyndromic hearing loss. Two hundred and one children with profound prelingual sensorineural hearing loss were included. Forty-six patients had 35delG in *GJB2*. Speech awareness thresholds (SATs) and speech recognition thresholds (SRTs) improved following implantation, but there was no difference in performance between patients with *GJB2*-related deafness versus control (all *P* > 0.10). Both groups had produced their first comprehensible words within the same period of time following implantation (2.27 months in *GJB2*-related deaf versus 2.62 months in controls, *P* = 0.22). Although our findings demonstrate the need to uncover unidentified genetic causes of hereditary deafness, they do not support the current policy for genetic screening before cochlear implantation, nor prove a prognostic value.

## 1. Introduction

Hereditary sensorineural hearing impairment (SNHI) affects 2–4 children per 1000 in the developing countries [1]. Burden of hearing impairment is described *in extenso* and is felt by the clinician and families taking care of the deaf [2]. Early identification of hearing loss and initiation of rehabilitation measures are the cornerstones of language development [3].

For patients with profound SNHI, cochlear implant (CI) is considered to be an effective intervention bypassing the inner ear organs and activating the auditory nerve directly. Outcome of CI differs significantly between the implantees. Since the etiology and demographic data of the implantees is diverse, different factors could be attributed to the difference of the CI success [4]. The operation is expensive and requires advanced training, therefore, identification of the most important factors affecting the outcome of CI becomes necessary especially in settings with limited resources and a need for judicious patient selection [5].

In the last two decades, molecular genetics had shed light on many aspects of hereditary deafness and elucidated the pathophysiology of deafness in some cases of genetic hearing loss. A number of studies had argued that *GJB2* mutations demonstrate a better language performance following cochlear implantation, but the justifications are not persuasive and authors frequently suggested further studies [6–9]. Since genetic testing is expensive and not covered by most healthcare insurances, identification of the role that genetics plays in the outcome of CI is far beyond necessary. In this study we investigated the role of commonly identified genetic mutations and their association with subjective and objective outcomes in a population of pediatric patients with profound hearing impairment that received CI. This population has a high rate of consanguineous marriage and therefore high rate of autosomal recessive diseases, which facilitated sampling nonsyndromic cases of congenital hearing loss [10, 11].

## 2. Methods

### 2.1. Subject Recruitment and Preimplantation Evaluation

Two hundred and one consecutive and unrelated children with profound SNHI (108 boys and 93 girls) who underwent CI from 2003 to 2006 were enrolled in this study. All children were native Persian speakers and had been identified to have hearing impairment before the age of 3, also known as prelingual hearing impairment. All of these children were found to be suffering from nonsyndromic hearing impairment, which means no additional physical problem had been identified accompanying their hearing loss. Comprehensive clinical evaluations were fulfilled for each child encompassing history, physical examination, audiological tests, radiological studies of the temporal bone, and genetic testing. These evaluations were done free of charge for all the candidates.

All the implantees had gone through 60 sessions of mandatory oral and language rehabilitation classes. These classes take 2 hours per session, and provide the minimum of the required time for sufficient tonal information of Persian language. All the parents and/or guardians of the implantees had previously signed a written consent for their implantation procedures and had exclusively signed a second consent for this study. The procedure and studies had been approved by the Institutional Review Board of Tehran University of Medical Sciences. 

### 2.2. Genetic Examination

Consenting subjects provided 7–10 cc of whole blood from which DNA was extracted using established techniques. Samples were screened for the 35delG allele of *GJB2* using a previously described ARMS-PCR method [12]. All 35delG homozygous samples were excluded from further testing. In the remaining samples, *GJB2* and *GJB6* mutation screening was completed using a previously described method [12].

### 2.3. Evaluation of Speech Perception

The date on which the CI recipient produced the first comprehensible two-syllable word was reported and confirmed by an audiologist. Data regarding the shape of audiogram and level of hearing was collected before the CI surgery for both ears and at 1, 3, 6, 12, and after 12 months from the date of CI for the implanted ear. None of the study subjects received bilateral cochlear implants. After implantation, speech awareness thresholds (SATs) were measured at 1 and 3 months and speech recognition thresholds (SRTs) were measured at 6 and 12 months. Most comfortable level (MCL) was also determined at 6 and 12 months. In some CI recipients, additional testing was done within the first 3 years following surgery. Although newer tests for assessment of auditory function exist, including the open-set and close-set tests, in the lack of standardized equivalent tests in Persian, we used level of hearing, SAT, SRT, and MCL to assess the outcome of CI and compare it between the two arms of the study. We should highlight that results from nonstandardized tests that are equivalent to tests in English language were all reproducing the same results but are excluded from the analysis for the sake of robust standard comparisons to other studies.

### 2.4. Statistical Analysis

SAT, SRT, and MCL were expressed in dB referring to the hearing level; speech recognition and word generation were compared to the genetic diagnosis. Continuous variables were compared using the Student's *t*-test, ANOVA, and repeated measures analysis; chi-square was used to compare dichotomous variables. All tests were two tailed. Differences were reported as significant if the *P* value was less than 0.05. Because age at the time of implantation could potentially confound the association between the genetic status and some of the primary outcomes (e.g., time of first vocalization), we used linear regression models to adjust the analyses for age. 

## 3. Results

Genetic analysis identified 62 patients with mutations in *GJB2*, and 35delG was the most frequent type of mutation. Forty-six patients were found to have 35delG mutation in *GJB2*: thirty (14.9%) were found to be homozygote for 35delG mutations and 13 (6.5%) were heterozygote; the second mutation was not found in the remaining three heterozygotes.  Table 1 lists the mutations found in the 16 patients without 35delG. No *GJB6* mutation was found in our population.

Ethnicity of our patients and the ethnicity of the Iranian population are shown in Table 2. In 124 families (61.7%) parents had been consanguineous relatives of each other. The mean age of implantation was 2.5 years (ranging from 8 months to 5.5 years). They were followed for an average of 3.72 years (standard deviation = 2.95 years), and their age at last data point ranged between 2 and 14 years (mean ± standard deviation = 6.38 ± 3.37 years). Duration of followup was not statistically different among patients with and without mutations (4.1 years versus 3.6 years, *P* = 0.234, 95% CI = −1.43 to 0.35 years, power > 99%).

Following implantation, audiograms improved from severe-to-profound or profound levels before the surgery to moderate-to-severe or moderate levels by 3 months after surgery, all acquiring a flat shape. By 6 months after surgery, all audiograms were at moderate level or better. Nearly half of implant recipients improved to mild hearing loss after one year following implantation (Table 3). Comparison of level of hearing among carriers of *GJB2* mutations versus control yielded no significant association (*P* > 0.10 in all comparisons).

At baseline, level of hearing had not been significantly different between the *GJB2* mutation carriers and the rest of implantees (*P* = 0.053, power = 96.65%, alpha level 0.05). In addition, there was no correlation between *GJB2*-related deafness and other demographic factors including race of patient or parents, education level, and occupational status of parents (ANOVA *P* > 0.10). Although level of hearing had improved over time, there was no statistically significant difference in this improvement between the carriers of *GJB2* mutations and the rest of the implantees (*P* = 0.18). Regression in each measured frequency of the audiograms over the period of followup did not show a significant difference between the *GJB2* mutation carriers and the rest of the patients (ANOVA *P* > 0.2, statistical power = 77.3%). The very same statistically similar findings were seen for the SAT, SRT, and MCL measurements at the specified times (all *P* > 0.10, statistical power > 70% in all cases). Results remained unchanged after correcting for age at the time of operation or age at the time of diagnosis.  Table 4 shows the measured SAT, SRT, and MCL values at the specified times, but further subcategorizes the group with *GJB2* mutations into two subgroups. The time of first vocalization did not significantly differ between the two groups (2.27 months for *GJB2*-related deaf versus 2.62 for controls, *P* = 0.22, statistical power = 90.4%). The association between *GJB2*-mutation and time of first vocalization remained insignificant after correction for age at the time of operation (*P* = 0.345) or age at the time of diagnosis (*P* = 0.215).

**Figure 1:**
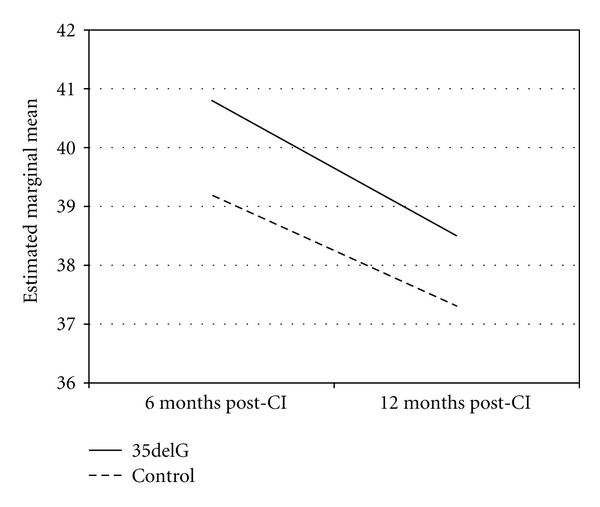
Changes in SRT following implantations in patients with 35delG mutation versus others with non-*GJB2*-related deafness.

**Figure 2:**
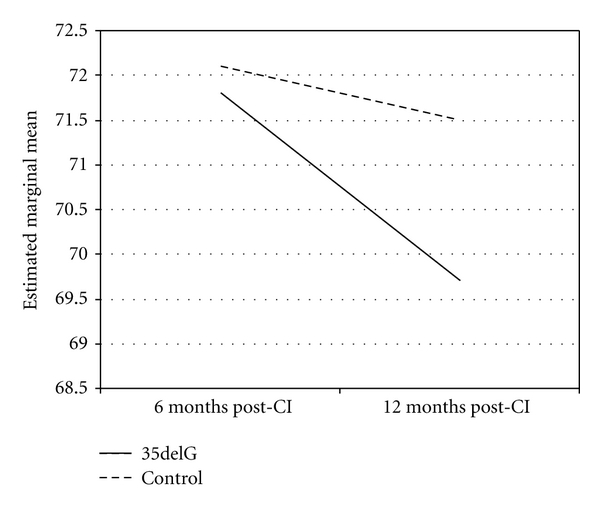
Changes in MCL following implantations in patients with 35delG mutation versus others with non-*GJB2*-related deafness.

Repeated measures analysis showed that although the level of hearing had significantly improved over time, the difference was independent of the genetic status of patients (*P* = 0.971). Similarly, presence of 35delG had no effect on the improvement observed in SRT (*P* = 0.973) or MCL (*P* = 0.511) over time. Figures 1 and 2 depict that the trend of improvement of these variables was similar in the two groups. We repeated this analysis by restricting it to patients with homozygote 35delG mutation, and yielded similar results: the presence of homozygote 35delG was not associated with improvements observed in SRT (*P* = 0.581) or MCL (*P* = 0.253). 

## 4. Discussion

This is the largest study to date on prelingual nonsyndromic deaf population investigating the effect of *GJB2* mutations on the outcome of cochlear implantation. We found that *GJB2* mutations do not have any significant relation with the speech perception abilities following CI compared to non-*GJB2*-related patients. In addition, no correlation was found between the genetic mutation and time to vocalization of the first comprehensible word. Other variables which had been shown to affect the outcome of CI such as age at implantation, duration of implant use, and residual hearing before CI were kept constant in this study through the patient selection criteria or at the time of analysis. In this regard, our studied population constitutes a relatively homogenous population to study the effect of genetic factors. Our study also shows that the genetic change in a large number of patients with hereditary SNHI remains unknown in our population.

At the time of clinical introduction of *GJB2* mutation screening for deaf patients, many came to the conclusion of *GJB2* superiority in CI recipients [9]. A primary study on the CI recipients had identified a better response in the carriers of mutations in *GJB2*. An issue raised about this study was patient selection and matched controls where a clear etiology of deafness in controls is not provided [13]. The same journal published another study in the following year which showed no benefit in the carriers of *GJB2* mutations [8]. This is the year when further studies had come to a conclusion of superiority of *GJB2* mutation carriers over non-*GJB2* controls [14]. The concept of *GJB2* performance superiority in CI performance was additionally reinforced within two years [7].

Correct selection of the matched controls for any deduction is emphasized in the works of other authorities [15]. More recent studies have been cautious in making any suggestion regarding the superiority of genetic deafness over controls for the CI outcome [16]. In 2008, a study on Taiwanese patients reported the same positive effect for genetic cases of hearing loss on the outcome of the CI. Although they had a very small number of *GJB2* patients and the larger proportion of identified genetic cases were due to SLC26A4, they proposed an intact auditory nerve as the cause of a better outcome in their implantees but had not studied or proposed other possible causes in the rest of their population. While a syndromic cause is usually identified following a physical examination, lack of the diagnosis in the rest of the recipients brings them into the category of other nonsyndromic causes of deafness that had not been tested. It that case, the theory of an intact auditory nerve could not be readily relied on [6]. One of the advantages of our study stems from the selection of nonsyndromic patients with a prelingual diagnosis of profound hearing loss, which makes the comparison easier by keeping more variables constant. Sampling nonsyndromic patients was particularly feasible in our study setting because of high rate of consanguinity of parents in the target population [10, 11].

Justification of a better auditory perception in genetic cases—including *GJB2* mutation carriers—by other authorities mainly lies in the intactness of the auditory nerve. In a population with such a high rate of consanguineous marriages and taking into consideration the high rate of deafness presenting as a nonsyndromic disease in general, correct selection of patients through physical examination, history taking, and required imaging modalities will choose a high rate of patients with intact auditory nerves. It should be highlighted that if only intact auditory nerve had been the support of justifications of a better response in congenital deafness, a much more simple way to choose such patients would have been through electrophysiological tests which are much less expensive compared to genetic tests.

In many articles which suggest a genetic effect on the outcome of CI, the studied population compromises a large variety of cases in which many syndromic deaf patients are also studied [7, 9, 13, 14]. This might have been a confounding factor in their final conclusion. The other question which remains to be answered by supporters of a positive genetic effect is where the problem had been in their patients with no identified genetic cause and still a functioning auditory nerve. The answer to this question might shed light on the reported effect of genetic hearing loss on the outcome of CI.

To the contrary, a perfectly matched, yet smaller study, on CI patients found that there is no advantage in connexin-associated deafness over other patients [17]. The same results had been reported through another two-center study earlier [18]. As indicated in our study, if the patients are selected through a delicate physical examination and other necessary investigations before CI, the acceptable outcome of the implantation would be assured. This will once again emphasize the importance of selecting a comparable control group in making a final conclusion [17]. The similarity of response to CI is also claimed in a recent study [19]. Very interestingly, a current publication on *GJB2* and CI outcome had found a poorer outcome following implantation in *GJB2* carrying patients [20]. All these data are self-explanatory of the debated genetic effect on the outcome of CI.

Although our initial aim was to evaluate the effect of *GJB2* and *GJB6* mutations on the outcome of CI in children with congenital hearing loss, our patient population appeared to have no *GJB6* mutation. Previous studies had reported *GJB6* mutations to be rare or absent in the Iranian population [21, 22]. Further studies on different patient populations might be done to assess whether our findings can be generalized to patients with *GJB6* mutations.

Our study is not without limitations. Similar to other studies discussed above, we are unable to clarify the pathophysiology of congenital hearing loss in the fraction with no *GJB2* mutation. Answering this question needs genome-wide studies which are beyond our scope. Additionally, because the collection of audiological data was based on manual review of medical records, we were unable to collect complete data on all patients. Finally, as mentioned previously, we used SAT, SRT, and MCL tests as our outcome measures due to a lack of speech-level tests standardized for Persian language. We suggest future researchers to validate open-set and close-set tests for Persian language and use them as their outcome measure.

In short, the findings of our study with a notable statistical power are not in accordance with the assumption that genetic mutations may be associated with different prognosis after CI. Given the intensive work and the expenses of CI with limited resources, particularly for countries where implants are used mainly for profoundly deaf children before the age of 3, we cannot support the hypothesis that genetic testing has a value to the CI procedure and its outcomes. When limited resources and lack of insurance coverage for the genetic tests are factored in, the costs of genetic testing can significantly overweigh its potential benefit. In the absence of larger studies with higher statistical power, a systematic review may enlighten the effect of mutations on the outcome of cochlear implantation and also investigate the reason for heterogeneous results of previous studies. 

## Figures and Tables

**Table 1:** *GJB2* genotypes in patients carrying no 35delG mutations. Homozygote mutations are marked with an asterisk.

Genotype	Frequency	Percent
V1531	4	25%
R184P*	2	12.5%
167DelT*	2	12.5%
V27I SNP*	2	12.5%
G130V	1	6.3%
235delC/R184P	1	6.3%
R127H	1	6.3%
SNP N26N*	1	6.3%
R32C*	1	6.3%
R151P*	1	6.3%

Total	16	100%

**Table 2:** Ethnicity of the studied population compared to overall composition in Iran.

Ethnicity	Number	Percentage	Percentage in Iran
Fars	68	33.8	51
Turk	44	21.9	24
Gilak/Mazandarani	34	16.9	8
Kurd	16	8.0	7
Lur	13	6.5	2
Arab	9	4.5	3
Pakistani	1	0.5	<1
Afghan	1	0.5	<1
Mixed	14	7.0	5 (other)
Not known	1	0.5	—

Total	201	100	100

**Table 3:** Percentages showing the shape of audiograms and level of hearing at the times measured.

Shape of audiogram	Before CI	1 mo	3 mo	6 mo	12 mo	12 mo <
Fragmentary	52.1	—	—	—	—	—
Descending	46.6	—	—	—	—	—
Flat	1.4	100	100	100	100	100

Level of hearing						
Profound	82.2	—	—	—	—	—
Severe-to-profound	17.8	—	—	—	—	—
Moderate-to-severe	—	13.9	2.8	—	—	—
Moderate	—	86.1	97.2	95.7	81.2	53.8
Mild	—	—	—	4.3	18.8	46.2

**Table 4:** SAT, SRT, and MCL measurements and gender distribution of patients in the sample. Patients with *GJB2* mutations are subcategorized into two columns, based on the presence of 35delG variant. Continuous values are presented as mean ± standard deviation; dichotomous values are presented as frequency (percentage in row). Values pertain to the operated ear only. Unit of measurement: dB.

		35delG mutation	Non-35delG mutation	No mutation
Gender	Male	26 (56.5%)	8 (50%)	74 (53.2%)
	Female	20 (43.5%)	8 (50%)	65 (46.8%)

SAT	*N* = 72	*N* = 20	*N* = 5	*N* = 47
	One month after surgery	43.6 ± 3.8	41.7 ± 2.9	44.1 ± 5.1
	Three months after surgery	40.7 ± 5.3	35.0 ± 0*	39.7 ± 4.8

SRT	*N* = 65	*N* = 19	*N* = 4	*N* = 42
	Six months after surgery	40.8 ± 4.5	43.3 ± 2.9	39.2 ± 4.7
	Twelve months after surgery	38.5 ± 4.7	37.7 ± 7.6	37.3 ± 4.1

MCL	*N* = 45	*N* = 13	*N* = 4	*N* = 28
	Six months after surgery	71.8 ± 4.0	75.0 ± 0.0*	72.1 ± 4.4
	Twelve months after surgery	69.7 ± 6.0	66.7 ± 7.6	71.5 ± 4.4

Total**		46	16	139

*All measured values were equal, hence a standard deviation equaling zero.

**Not all values presented in the table were available for the total number of subjects in each group.
